# Characterizing Spatio-Temporal Variation in Macroinvertebrate Communities and Ecological Health Assessment in the Poyang Lake Basin During the Early Stage of a Fishing Ban

**DOI:** 10.3390/ani15162440

**Published:** 2025-08-20

**Authors:** Chunhua Zhou, Ruobing Zhao, Wenxin Xia, Fangfa Zeng, Yanqing Deng, Wenhao Wang, Shan Ouyang, Xiaoping Wu

**Affiliations:** 1 School of Life Sciences, Nanchang University, Nanchang 330031, China; zhouchunhua@ncu.edu.cn (C.Z.); zhao741211@163.com (R.Z.); lars1122@163.com (W.X.); 13563066859@163.com (W.W.); liminghuadi@163.com (S.O.); 2 Jiangxi Province Key Laboratory of Ecohydrological Monitoring Research in Poyany Lake Basin, Nanchang 330031, China; 3 Jiangxi Ecological Environment Monitoring Center, Nanchang 330019, China; zeng19870910@126.com; 4 College of Water Conservancy & Hydropower Engineering, Hohai University, Nanjing 210098, China; 5 Monitoring Center on Hydrology and Water Resources in the Lower Reaches of the Ganjiang River, Yichun 336000, China

**Keywords:** macroinvertebrate, diversity, ecological health, Poyang Lake, early stage of the fishing moratorium

## Abstract

Macroinvertebrates are a crucial part of aquatic ecosystems and significantly contribute to the maintenance of their health and stability. Our aims were to explore spatio-temporal patterns in macroinvertebrate communities and evaluate the ecological health of various parts of the Poyang Lake Basin during the early stage of a fishing ban. We collected samples using a Peterson grab sampler and conducted ecological evaluations using the B-IBI index. A total of 107 species of macroinvertebrates were identified, and most species were arthropods. The density and biomass of macroinvertebrates significantly differed among seasons and water bodies. No significant differences in diversity among seasons were observed; however, diversity significantly varied among water bodies. Environmental parameters such as water depth, pH, turbidity, total nitrogen, total phosphorus, and chlorophyll a played a crucial role in shaping the community structure of macroinvertebrates. Most of the sampling sites were classified as healthy or sub-healthy, indicating that the fishing ban policy has started to have a positive effect. The effects of this ban are achieved through a cascading sequence of processes, including the elimination of fishing disturbance, the restoration of habitat structure, and the reallocation of trophic energy, in addition to increases in microhabitat diversity associated with habitat heterogeneity. Together, these processes drive the multidimensional recovery of macroinvertebrate communities, manifested as increased species richness, higher density and biomass, and elevated B-IBI scores.

## Simple Summary

We sampled macroinvertebrates from six areas in China’s largest lake during the early stage of a fishing ban. We found a wide variety of species and noticed that their density and biomass generally rose. Habitat heterogeneity has a significant impact on macroinvertebrates. They are sensitive to multiple environmental physical parameters. We evaluated the ecological health of these six water areas using the B-IBI index. This implies that the implementation of the fishing ban policy is conducive to the restoration of ecological health. The removal of fishing disturbance and the restoration of habitat structure could be possible reasons for obtaining such results after the fishing ban.

## 1. Introduction

Macroinvertebrates are a vital component of aquatic ecosystems [1]. They are key intermediate links in aquatic food webs and facilitate both energy flow and nutrient cycling within aquatic systems [2]. Macroinvertebrates are widely distributed in rivers, have weak migration capacities, and have long life cycles [3]. The number of macroinvertebrate species has been used as an indicator in assessments of the health of rivers and lakes. Because macroinvertebrates are highly sensitive to environmental changes, their community structures typically respond to environmental disturbances in a predictable manner. The disappearance of sensitive species and the dominance of tolerant taxa such as chironomids and oligochaetes signal a shift to a “low-diversity, low-stability” state, in which energy cycling and sediment-stabilizing functions are severely impaired. Therefore, they are considered important indicators for evaluating the health of aquatic ecosystems [4]. The Benthic Macroinvertebrates Index of Biotic Integrity (B-IBI) for health assessment can provide a relatively comprehensive reflection of the overall condition of aquatic ecosystems, as well as changes and trends in their habitats [5]. The B-IBI has been widely used for assessing aquatic ecosystem health [6].

Poyang Lake is situated in northern Jiangxi Province and is China’s largest freshwater lake, covering an area of approximately 3409 km^2^. The seasonal variation in water flow of the lake is typical for lakes in the region [7]. The average annual rainfall is approximately 1500 mm, which is mainly concentrated in spring and summer [8]. It carries the water from important rivers and their tributaries in Jiangxi Province, which makes it a hydrogeographically significant region [9]. The ecosystem of Poyang Lake is unique, the habitats are complex and diverse, the aquatic biodiversity of the lake is rich, and the seasonal variation in water flow is typical for lakes in the region. Poyang Lake, one of the major lakes freely connected to the Yangtze River, is crucial for maintaining the security of the aquatic ecosystem in the middle and lower reaches of the Yangtze River [10]. However, decades of overfishing have degraded the ecosystem and altered the structure of aquatic communities, which has pushed several species to the brink of extinction. A fishing moratorium is therefore needed to break this cycle and restore aquatic biodiversity. Global “lockdown/fishing ban” experiences have repeatedly demonstrated that reducing human disturbance—even for a short period—can rapidly lower pollution loads in aquatic systems and open a critical window for biodiversity recovery [11,12]. Since January 1, 2020, fishing bans have been implemented in critical water areas of the Yangtze River Basin. Before the fishing ban, the macroinvertebrates and ecological health of Poyang Lake received considerable research attention [13,14,15,16,17,18,19,20]. However, studies have been scarce since the moratorium was enacted.

Here, we examined the hypothesis that the fishing ban will enhance macroinvertebrate diversity. We conducted an ecological investigation of the aquatic macroinvertebrates in Poyang Lake Basin in the spring, summer, autumn, and winter of 2021. The aims of this study were to (1) clarify the diversity and community structure of macroinvertebrates and their relationships with major environmental parameters and (2) evaluate ecological health based on the IBI of macroinvertebrates (B-IBI). Our findings will aid the protection and management of aquatic organisms after the fishing ban and provide fundamental information for assessing the impact of the decade-long fishing moratorium in the Yangtze River.

## 2. Materials and Methods

### 2.1. Sample Collection

**Figure 1:**
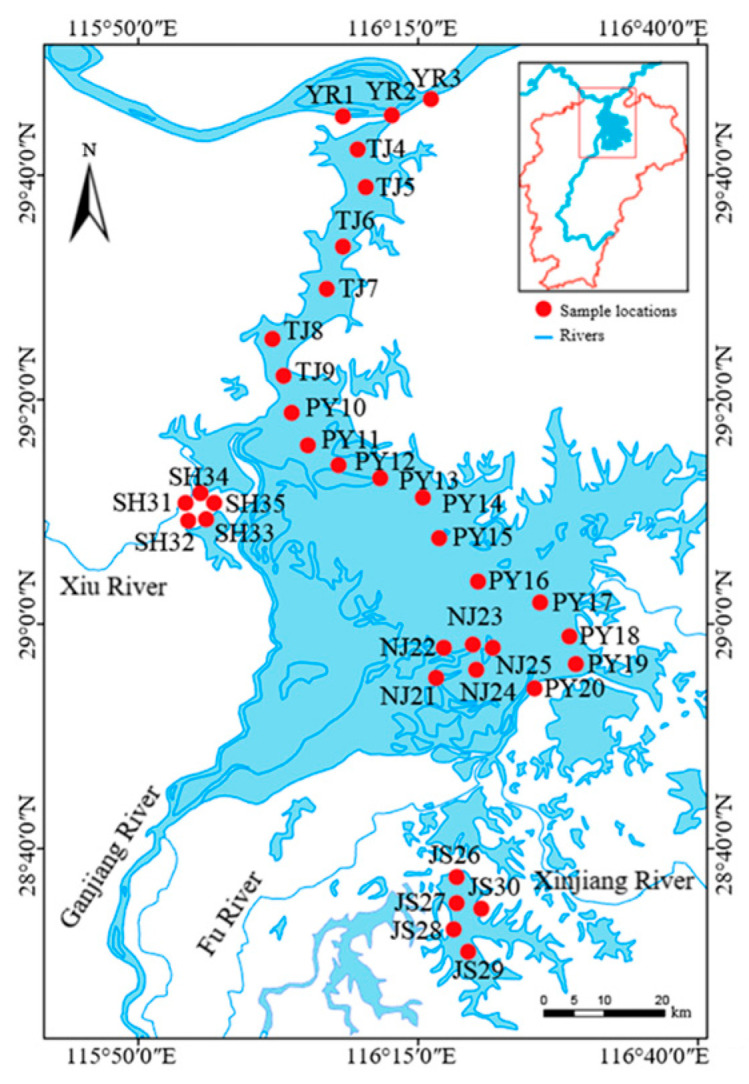
Sampling sites in this study. YR, Jiangxi section of the main stream of the Yangtze River; TJ, the channel connecting Poyang Lake and the Yangtze River; PY, the main lake area of Poyang Lake; NJ, Nanjishan Nature Reserve; JS, Junshan Lake; SH, Sha Lake.

The sampled waters were divided into six areas based on their characteristics, including the Jiangxi section of the main stream of the Yangtze River (YR), the channel connecting Poyang Lake and the Yangtze River (TJ), the main lake area of Poyang Lake (PY), Nanjishan Nature Reserve (NJ), Junshan Lake (JS), and Sha Lake (SH). A total of 35 sampling points were established in the study area (Figure 1). Quantitative collection of macroinvertebrates was carried out at each sampling site in January (winter), April (spring), July (summer), and October (autumn) 2021. A Peterson grab sampler (1/16 m^2^) was used to collect sediment samples, and three replicates were collected at each sampling location. The sediment samples were washed through a 40-mesh sieve and then poured into a white porcelain dish to select macroinvertebrates. The specimens were placed in a 10% formaldehyde solution for fixation. Qualitative collection was carried out using a hand screen or via manual examination. The specimens were brought to the laboratory for classification and identification. The quantities of various types of specimens were determined. After absorbing the surface moisture of the specimens with absorbent paper, they were weighed (accuracy of 0.01 g). The density and biomass per square meter were calculated. While sampling, water depth (WD) was measured. Flow velocity (V) was determined using a current meter. The water temperature (T), turbidity (Turb), pH, salinity (Sal), chlorophyll-a (Chl-a), and dissolved oxygen (DO) were assessed using a YSI6600V2 multi-parameter water quality monitor. Two L of water was collected at each sampling site with a water sampler and transported to the laboratory for the determination of total nitrogen (TN) and total phosphorus (TP).

### 2.2. Data Analysis

One-way ANOVA was performed to test the significance of the effects of environmental parameters in the sites sampled. The mean ± standard deviation was used to describe the environmental parameters in each area. Principal component analysis (PCA) was used to compare differences in environmental parameters before and during the ban period. Adonis permutational multivariate analysis of variance (PERMANOVA) was used to assess the significance of differences. The environmental parameters of the pre-ban period were derived from our previously published work [9]. The dominant species in each sampling area were determined using the dominance degree value $Y=\left(\frac{N_{i}}{N}\right)\times F_{i}$, where *Ni* represents the number of individuals of the *i*-th species, *N* represents the total number of individuals, and *Fi* represents the frequency of occurrence of the *i*-th species. When the dominance value Y was greater than 0.02, the species could be classified as the dominant species for further analysis. The Richness index (R), Shannon–Weiner index ($H^{\prime }$), and Pielou index ($J^{\prime }$) were used to characterize the alpha diversity of macroinvertebrates. The formulas for calculating indices were as follows:

$$R=\frac{S-1}{{log}_{2}N}$$

$$H^{\prime }=-\sum_{i=1}^{S}P_{i}{Iog}_{2}P_{i}$$

$$J^{\prime }=H^{\prime }/{log}_{2}N$$

where Pi represents the proportion of individuals of species i relative to the total number of individuals, and S is the total number of species in the sample. Non-metric multi-dimensional scaling analysis (NMDS) was conducted based on the Bray–Curtis distance matrix, and differences in the community structure of macroinvertebrates were determined through similarity analysis (ANOSIM). Species beta diversity was studied by analyzing its two components, species turnover (βsim) and nestedness (βsne), following the method of Carvalho et al. [21]. We used the abundance biomass comparison (ABC) curve to analyze whether the communities in the six water sampling areas were affected by environmental disturbances. The ABC curve was plotted using PRIMER 5.0 software. The statistical quantity of the ABC curve is represented by the W-value. When the W-value is positive, the biomass curve is above the abundance curve, indicating no disturbance; when the W-value is close to 0, the two curves are close to each other or partially intersect, indicating moderate disturbance; and when the W-value is negative, the biomass curve is below the abundance curve, indicating severe disturbance. Redundancy analysis (RDA) was conducted on the relationship between macroinvertebrate communities and environmental factors.

### 2.3. Biological Integrity Evaluation System for Macroinvertebrates

Reference points and disturbed points were designated. The following evaluation criteria were used to classify reference sampling points and disturbed sampling points per a previous study [22] (Table 1).

A total of 31 candidate indicators (Appendix A) were used based on previously described classification methods [23,24]. Next, the candidate indicators were screened. Indicators that differed by less than 10% among all sampling points and indicators with index values that were 0 in more than 90% of all sampling points were removed. Indicators wherein the medians of the reference point and the observation point were within the quantile range of 25% to 75% of each other were also removed. Correlation analysis was conducted on the remaining indicators. If the correlation coefficient between two indicators was greater than 0.75, one of the indicators with more information was used. Finally, the evaluation indicators were obtained.

The ratio method [25] was used to score and calculate B-IBI in the Poyang Lake Basin. The B-IBI values of each sampling point were obtained by adding up the scores of each evaluation indicator. The 25% percentile of the total B-IBI score at the reference point was used as the health threshold. Scores below this threshold were evenly divided into four groups to evaluate the health status: sub-healthy, moderate, poor, and extremely poor.

## 3. Results

### 3.1. Environmental Factors

**Figure A1:**
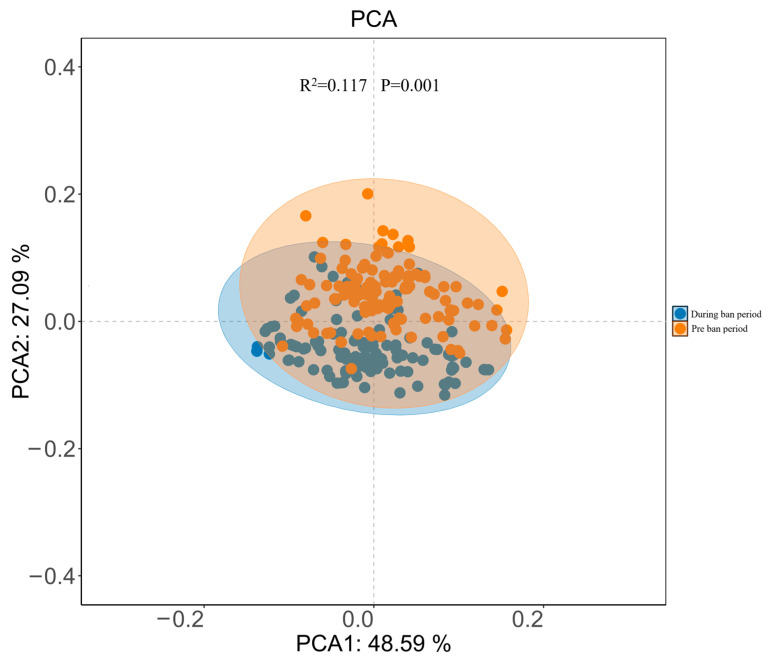
PCA of environmental parameters before and during ban period.

Analysis of differences in environmental parameters revealed significant disparities among the various sampling areas for all environmental parameters, with the sole exception of water temperature (Table 2). YR had the greatest flow velocity, the greatest water depth, and high annual average pH and salinity. The turbidity was highest in the waters of NJ and lowest in JS. SH had the highest annual average dissolved oxygen, Chl-a, and total phosphorus (Table 2). PCA (Appendix A: Figure A1) revealed a highly significant difference in environment parameters before and during the ban period (R^2^ = 0.117, *p* = 0.001).

### 3.2. Species Composition and Dominant Species

We identified 107 different species of macroinvertebrates, and these belonged to 3 phyla, 7 classes, 32 families, and 78 genera. Arthropods were the most diverse, comprising 64 species, which represented 59.8% of the total species identified. Mollusks accounted for 20.6% of the total species, with 22 species identified, and annelids comprised 19.6% of the total species, with 21 species recorded. A total of 45 species were identified in spring, 39 in summer, 48 in autumn, and 49 in winter. A total of 58 and 53 species were identified in PY and NJ, respectively. TJ, SH, and JS had 35, 29, and 25 species, respectively. The minimum number of species in YR was 11.

There was a total of 11 dominant species of macroinvertebrates in the six sampling areas, including 3 species of annelids, 3 species of mollusks, and 5 species of arthropods. The dominant species varied among sampling areas. *Gammarus* sp. was the only dominant species in YR. Junshan Lake had the most dominant species, and all of these were species with high pollution tolerance (Table 3).

### 3.3. Spatio-Temporal Variation in Density and Biomass

**Figure 2:**
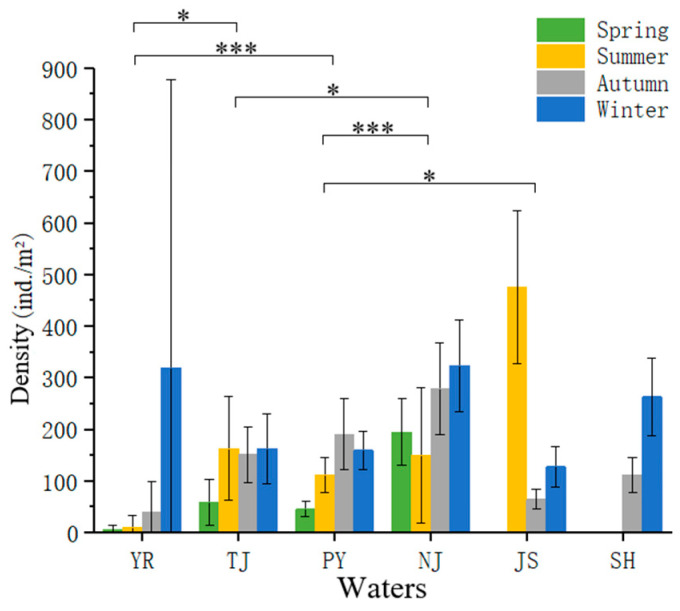
Spatio-temporal variation in the density of macroinvertebrates. The vertical lines represent the size of the standard error. * *p* < 0.05; *** *p* < 0.001.

The annual average density of macroinvertebrates in the Poyang Lake Basin was 165.88 ± 92.20 ind./m^2^, and the average biomass was 146.28 ± 130.25 g/m^2^. Density varied significantly among seasons (*p* < 0.05). The average density was highest in January (winter), at 213.98 ± 113.89 ind./m^2^, and lowest in April (spring), at 86.32 ± 47.68 ind./m^2^ (Figure 2). The density varied significantly among water sampling areas (*p* < 0.001). The average density was highest in the Nanjishan Nature Reserve water area (237.22 ± 91.04 ind./m^2^) and lowest in YR (94.67 ± 305.68 ind./m^2^) (Figure 2). Biomass varied significantly among seasons and water bodies.

### 3.4. Diversity and Community Structure

**Figure 3:**
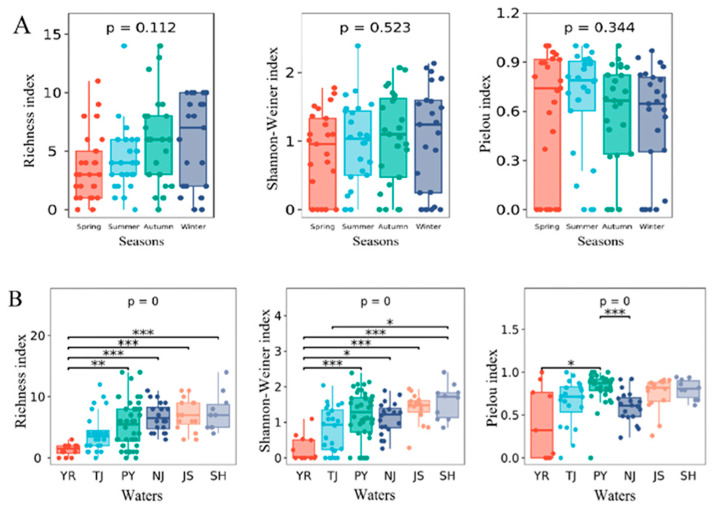
Alpha diversity in the different seasons (**A**) and water sampling areas (**B**). * *p* < 0.05; ** *p* < 0.01; *** *p* < 0.001.

In the Poyang Lake Basin, no significant seasonal differences were observed in the alpha diversity index (Figure 3A); however, pronounced spatial heterogeneity in the alpha diversity index was observed (Figure 3B). The average Richness index was lowest in YR, followed by TJ (Appendix A). The Richness index was significantly lower in YR than in PY, NJ, JS, and SH (*p* < 0.05) (Figure 3B). The same pattern was also observed in the Shannon–Weiner index. The average Pielou index was lowest in YR and highest in PY (Appendix A). Significant differences were observed between these two sampling areas (*p* < 0.05).

**Figure 4:**
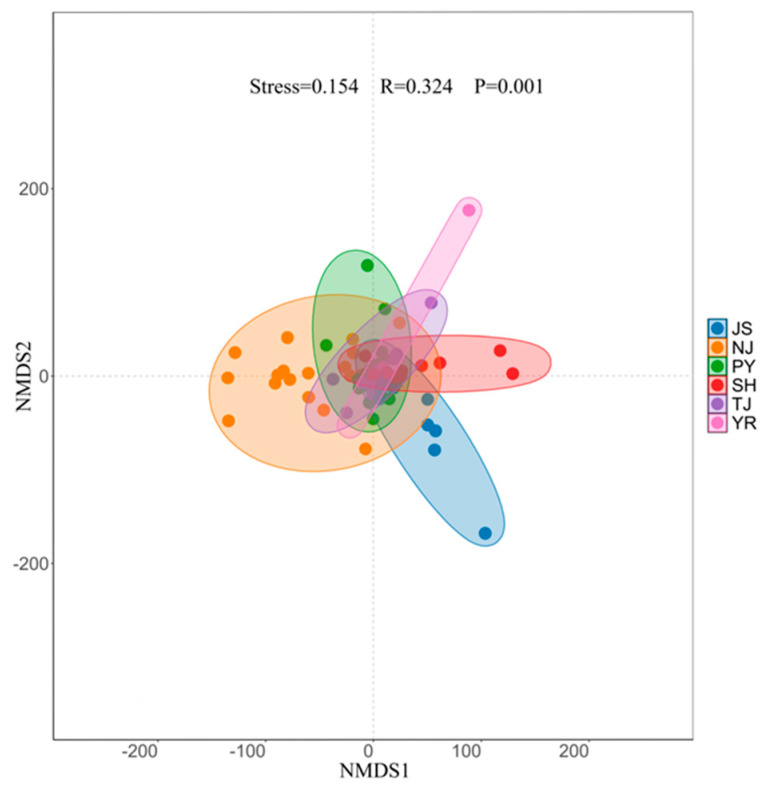
NMDS analysis of macroinvertebrate communities.

NMDS analysis of the composition of macroinvertebrates in the six water sampling areas yielded a stress value < 0.2 and R-value > 0, indicating that the grouping was meaningful (Figure 4). The overall *p*-value was less than 0.01, indicating that there were extremely significant differences in the composition of macroinvertebrate communities among the grouped samples, and these differences were observed across all pairwise water sampling areas, with the exception of TJ and PY (Figure 4).

**Figure 5:**
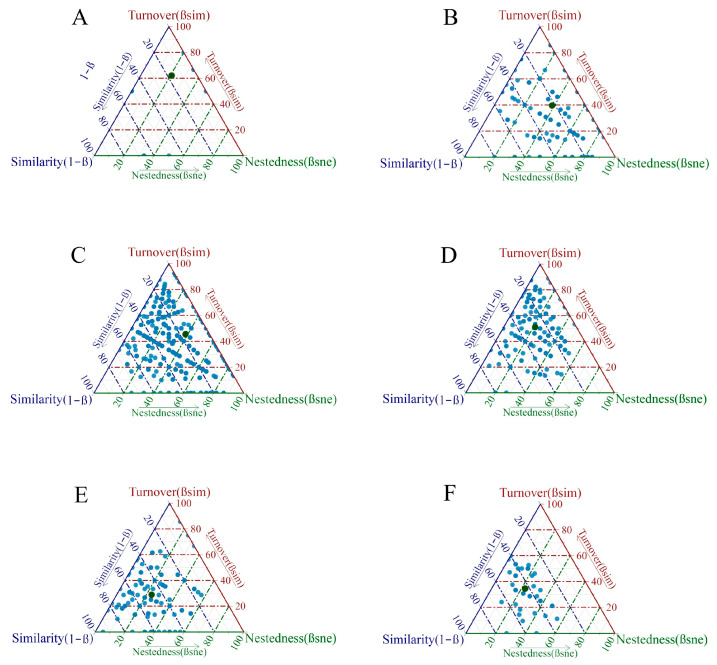
Beta diversity in different sampling areas ((**A**) YR; (**B**) TJ; (**C**) PY; (**D**) NJ; (**E**) JS; (**F**) SH).

Beta diversity analysis showed that beta diversity was high in PY, followed by YR and TJ. The turnover components in each water area were all greater than the nestedness components. The average contribution of the turnover components of beta diversity was highest in YR (74.86%), followed by NJ (70.78%). The proportions of the turnover components for both YR and NJ were much greater than the proportions of the nestedness components (Table 4, Figure 5).

### 3.5. Evaluation of Community ABC Curves

**Figure 6:**
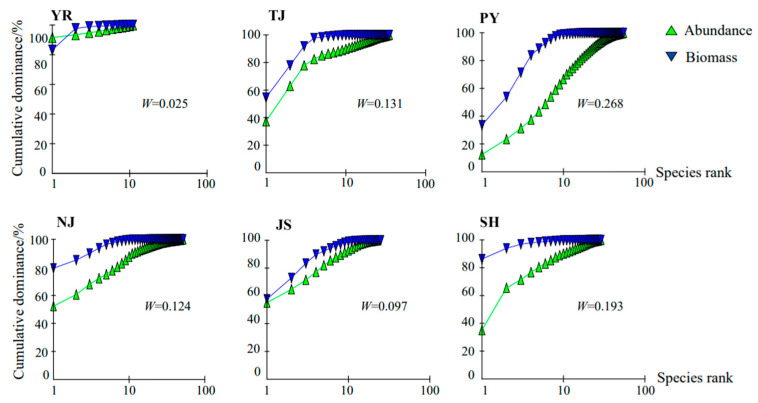
Spatial differences in the ABC curve of macroinvertebrates.

The ABC curves of different water sampling areas indicated that the biomass curve and abundance curve of YR and the W-value were close to 0. According to the relationship between the W-value and the disturbance of the community, the macroinvertebrate community was moderately disturbed and not stable. The biomass curves of the remaining water sampling areas were all above the abundance curves; the macroinvertebrate communities were thus not disturbed and were relatively stable (Figure 6).

### 3.6. Redundancy Analysis of Environmental Factors and the Community Structure of Macroinvertebrates

**Figure 7:**
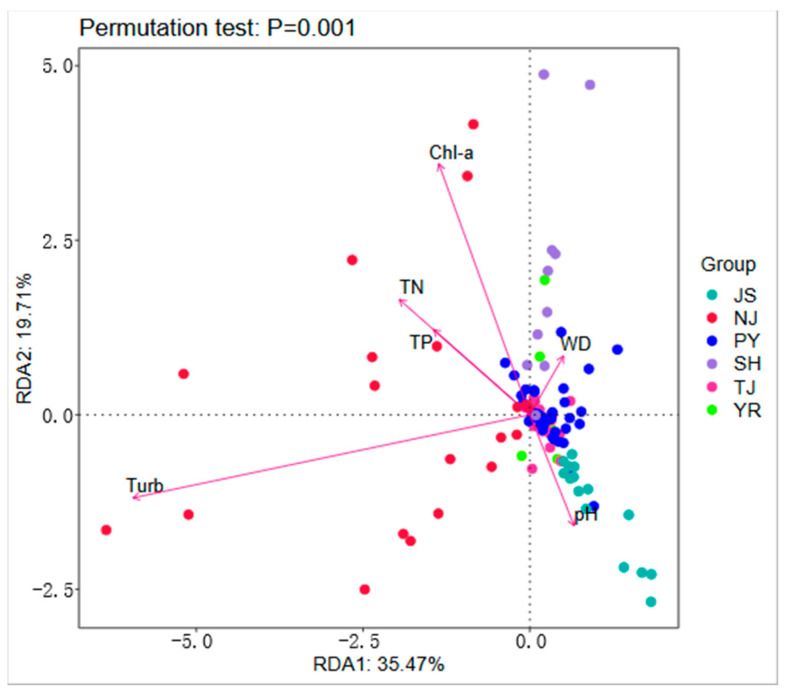
RDA of the macroinvertebrate community and environmental factors.

Correlation analysis of environmental factors showed that WD, Turb, pH, Chl-a, TN, and TP all significantly affected macroinvertebrate community structure. Therefore, in the RDA analysis, only environmental factors that significantly affected the ranking results were used. Permutation tests indicated that environmental factors significantly influenced the structure of macroinvertebrate communities (*p* < 0.01; Figure 7).

### 3.7. Screening and Establishment of Biological Integrity Indicators

A total of 14 reference sampling points and 24 disturbed sampling points were designated according to the evaluation criteria. M2, M4, M5, M6, M9, M11, M12, M13, M16, M21, and M23 were removed from the 31 candidate indicators after screening. The discriminative ability of the remaining indicators was assessed. M1, M7, M8, M15, M17, M19, M20, M22, and M28 met the screening requirements and advanced to the next round of screening. Spearman correlation coefficient analysis was conducted on these nine candidate indicators (Table 5). The indicators with correlation coefficients greater than 0.75 were removed, and four evaluation indicators were obtained (M1, M7, M20, and M22).

### 3.8. Scoring and Evaluation

The scores of the four evaluation indicators were calculated using the ratio method, and the cutoff values of the five evaluation criteria were 2.07, 1.55, 1.04, and 0.52 (Table 6). The B-IBI results showed that most of the 35 sampling sites were classified as healthy or sub-healthy.

There were 13 healthy sampling sites and 10 sub-healthy ones; 65.71% were either healthy or sub-healthy. There were eight moderate sampling sites, accounting for 22.86%. There were two poor and two extremely poor sampling sites. YR3 was healthy, YR1 was poor, and YR2 was extremely poor. Among the six sampling points in TJ, one was healthy (TJ9), and five were moderate. Among the 11 samples in PY, PY16 and PY18 were poor and extremely poor, respectively; the rest were healthy and sub-healthy. The waters of NJ and JS were healthy or sub-healthy. The two sampling points SH32 and SH34 in Sha Lake were moderate (Table 7).

## 4. Discussion

### 4.1. Characteristics of the Macroinvertebrate Communities

The resources of macroinvertebrates in Poyang Lake have decreased over time [16]. This might be related to the intensification of human activities and environmental alterations in Poyang Lake caused by natural factors, such as the decrease in water level, reductions in aquatic plants, and sand mining. However, our findings indicate that the number of species (107/58), density (165.88/134.88), and biomass (146.28/121.83) of macroinvertebrates in PY have increased since the fishing ban compared with a recent study conducted prior to this ban [16]. This indicates that the implementation of a ten-year fishing ban could aid the restoration of biodiversity. The dominant species in this study were *Corbicula fluminea*, snails, and species with higher pollution tolerance (Table 3). The community structure of macroinvertebrates in Poyang Lake has changed significantly over time. The dominant species evolved into small mollusks and pollution-tolerant Chironomidae and Oligochaeta [17]. This indicates that mussel resources are gradually declining. The density of benthic animals has also been shown to decrease in Dongting Lake, and more pollution-tolerant species have become dominant [26].

### 4.2. Macroinvertebrate Diversity and Community Structure

No seasonal differences in the alpha diversity of macroinvertebrates were observed (Figure 3A). However, a survey in 2016 showed seasonal differences [16]. This might stem from variation in water bodies among studies. Significant spatial variation was observed (Figure 3B). The alpha diversity of YR was the lowest because of the small number of species and the absolute dominance of *Gammarus* sp. Extremely significant differences were observed in the community structure of macroinvertebrates in different water areas (Figure 4). Significant differences in the composition of macroinvertebrate communities have also been observed in other biological systems [9,27].

Beta diversity was high in YR and PY (Table 4, Figure 5). This indicates that the species turnover rate among their macroinvertebrate communities was high or there were few common species. The turnover components of each water area were all greater than the nestedness components. This was the case not only in the lake area but also in the rivers [16]. The turnover components in both YR and NJ were much larger than the nestedness components (Table 4, Figure 5). This reflected the high heterogeneity and complexity in species composition among communities. This may be related to the significant differences in environmental conditions, restrictions on species diffusion, or the differentiation of ecological niches. The macroinvertebrate community of YR was moderately disturbed and unstable (Figure 6), which was related to its low alpha diversity and high beta diversity. The microhabitats within a site were uniform, and only tolerant species survived (low alpha diversity); however, there were significant environmental differences among microhabitats, which resulted in differences in species composition (high beta diversity).

### 4.3. Effects of Environmental Factors on Macroinvertebrates

Environmental factors directly reflect the suitability of habitat for macroinvertebrates, and they are closely related to the composition and assembly of macroinvertebrate communities [28]. The flow velocity of the regional water environment, the substrate environment, and the state of the riverbank affect macroinvertebrate communities [29,30]. In the waterway area, the water level is deep, and the bottom mainly comprises sand and gravel; sand mining activities have had a major effect on the waterway area. The results of this study indicate that the environmental factors in different water sampling areas of Poyang Lake varied greatly; the composition, biomass, and density of macroinvertebrate species in the six water sampling areas also varied significantly (Table 2, Figure 2). This indicates high habitat heterogeneity in the Poyang Lake Basin. Due to the high flow velocity, muddy bottom, and water depth, the number of macroinvertebrate species and biomass of macroinvertebrates were lower in YR than in other water sampling areas (Figure 3B). Macroinvertebrates with streamlined body types that are strong swimmers can better adapt to environments with faster flow rates [31]. In contrast, oligochaetes are more suited to habitats with lower flow rates [32].

RDA of environmental factors and the community structure of macroinvertebrates indicated that WD, Turb, pH, Chl-a, TN, and TP significantly affect community structure. Excessively deep water levels affect the photosynthesis of organisms by influencing the intensity of light and reducing the light transmittance of water bodies, thereby reducing macroinvertebrate food sources [33]. When the water level is too shallow, the risk of exposure to predators increases for macroinvertebrates [34]. The pH and turbidity of water bodies also affect the structure of macroinvertebrate communities [35]. The pH significantly influences the reproductive capacity of macroinvertebrates. A pH value less than 5 leads to a low birth rate in macroinvertebrates [36]. Turbidity is negatively correlated with transparency and affects the composition of food for macroinvertebrates [37,38]. Indicators such as total nitrogen and total phosphorus describe the content of different forms of nitrogen and phosphorus salts in the water environment. These indicators can reflect the overall status of nutrients in the water environment, thereby affecting primary productivity [39]. The concentration of Chl-a reflects the biomass of phytoplankton and algae, which are important food sources for many filter-feeding macroinvertebrates [40]. These factors affect macroinvertebrates through direct or indirect ecological pathways.

### 4.4. Health Evaluation of the Poyang Lake Basin

Before the 21st century, the PSR model indicated that the Poyang Lake Wetland was in an unhealthy state [18]. After the 21st century, the ecological environment has gradually improved due to the implementation of the ecological project of returning farmland to wetlands. Before the fishing ban, the ecological health of PY was moderate, and that of YR was poor according to the B-IBI index [19,20]. The findings of this research indicate that 72.72% of the sampling points in PY were healthy or sub-healthy, and there were no poor or extremely poor points in TJ. This indicates that the aquatic environment of the Poyang Lake Wetland has shown signs of improvement, suggesting that the fishing ban has had a positive impact. Therefore, the fishing ban policy should be continued. It is now unequivocally clear that restricting human activity in any region rapidly improves environmental quality and drives a measurable upturn in biodiversity—a pattern observed worldwide during the 2020–2022 lockdowns [12].

## 5. Conclusions

During the initial stage of the fishing ban, the number of macroinvertebrate species, as well as the density and biomass of macroinvertebrates, increased in Poyang Lake. The high habitat heterogeneity contributes to significant differences in environmental factors, alpha diversity, and community structure among water sampling areas. The turnover components of beta diversity were more important than the nestedness components. Multiple environmental factors significantly affected the species composition of macroinvertebrates. The evaluation using B-IBI demonstrated that the health of the aquatic ecosystem in the Poyang Lake Basin has improved. We recommend maintaining the fishing moratorium and strengthening scientific monitoring efforts.

## Figures and Tables

**Table 1:** The criteria for classifying a sampling point as a reference sampling point and a disturbed sampling point.

Screening Criteria	Reference Sampling Point	Disturbed Sampling Point
Shannon–Wiener diversity index (H′)	H′ ≥ 3	H′ < 3
Human disturbance activities	Minimal or no disturbance	Strong disturbance
Vegetation coverage	High vegetation cover, predominantly non-agricultural	Severe vegetation degradation, dominated by agricultural vegetation
Human residents	No human residents	Presence of human residents

**Table 2:** Water environment parameters of different water samples in Poyang Lake. Different letters in the table indicate significant differences in environmental parameters between water sampling areas (*p* < 0.05) by one-way analysis of variance.

Parameter	YR	TJ	PY	NJ	JS	SH
WD (m)	12.49 ± 10.19a	10.33 ± 4.78a	6.5 ± 4.5b	4.23 ± 1.95b	4.68 ± 1.4b	11.53 ± 8.8a
V (m/s)	0.38 ± 0.2ab	0.32 ± 0.18a	0.21 ± 0.12b	0.1 ± 0.02c	0.15 ± 0.03c	0.11 ± 0.22c
Turb (NTU+)	13.5 ± 6.99bc	25.78 ± 25.12b	9.94 ± 8.85c	71.33 ± 50.90a	8.35 ± 6.11bc	10.27 ± 7.14c
T (°C)	19.4 ± 5.63a	19.69 ± 8.40a	20.58 ± 8.11a	20.63 ± 0.61a	19.16 ± 9.61a	17.6 ± 8.61a
pH	7.86 ± 0.19a	7.55 ± 0.33b	7.31 ± 0.60b	7.00 ± 0.43c	7.41 ± 0.47b	6.74 ± 0.62c
Sal (mg/L)	0.14 ± 0.07a	0.06 ± 0.02bc	0.04 ± 0.02c	0.06 ± 0.11b	0.05 ± 0.04bc	0.03 ± 0.01c
DO (mg/L)	9.29 ± 0.28b	9.79 ± 0.45b	9.8 ± 2.00b	9.72 ± 1.60b	10.15 ± 0.48b	11.94 ± 1.61a
Chl-a (μg/L)	2.49 ± 3.57c	1.56 ± 0.91cd	2.06 ± 1.31cd	6.51 ± 4.16b	0.85 ± 0.42d	15.18 ± 4.44a
TN (mg/L)	2.58 ± 0.41b	2.21 ± 0.41a	2.2 ± 0.55a	2.45 ± 1.11a	1.26 ± 0.2a	2.51 ± 1.35a
TP (mg/L)	0.14 ± 0.05bc	0.13 ± 0.07c	0.13 ± 0.04c	0.08 ± 0.09a	0.07 ± 0.02d	0.18 ± 0.08ab

**Table 3:** The dominant species of macroinvertebrates in different water sampling areas of Poyang Lake.

Dominant Species	Degree of Dominance					
	YR	TJ	PY	NJ	JS	SH
*Nephtys oligobranchia*	—	0.15	0.04	—	0.05	—
*Tubifex sinicus*	—	—	—	—	0.02	—
*Branchiura sowerbyi*	—	—	—	—	0.06	—
*Bellamya purificata*	—	0.07	0.07	0.5	—	0.05
*Parafossarulus eximius*	—		0.02	0.05	—	—
*Corbicula fluminea*	—	0.03	0.02	—	—	—
*Gammarus* sp.	0.38	0.16	—	—	—	—
*Chironomus sinicus*	—	—	—	—	0.47	—
*Clinotanypus* sp.	—	—	—	—	0.05	—
*Tanypus punctipennis*	—	—	—	—	0.02	0.35
*Ceratopogonus* sp.	—	—	—	—	—	0.3

**Table 4:** Spatial differences in the beta diversity of macroinvertebrates.

Waters	Βsor	Βsim	βsne	βsim%	βsne%
YR	0.829	0.620	0.208	74.86	25.14
TJ	0.778	0.395	0.383	50.78	49.22
PY	0.838	0.454	0.384	54.18	45.82
NJ	0.722	0.511	0.211	70.78	29.22
JS	0.524	0.289	0.235	55.10	44.90
SH	0.570	0.345	0.225	60.49	39.51

**Table 5:** Results of redundancy analysis of candidate indicators. * *p* < 0.05; ** *p* < 0.01.

	M1	M7	M8	M15	M17	M19	M20	M22	M28
M1	1								
M7	0.665 **	1							
M8	0.656 **	0.983 **	1						
M15	0.469 **	0.834 **	0.811 **	1					
M17	0.845 **	0.686 **	0.689 **	0.452 **	1				
M19	0.874 **	0.730 **	0.736 **	0.486 **	0.967 **	1			
M20	−0.552 **	−0.384 **	−0.390 **	−0.154	−0.762 **	−0.670 **	1		
M22	0.692 **	0.500 **	0.492 **	0.318 *	0.692 **	0.687 **	−0.482 **	1	
M28	0.516 **	0.822 **	0.796 **	0.977 **	0.495 **	0.524 **	−0.198	0.363 **	1

**Table 6:** Criteria used to assess aquatic ecosystem health in the Poyang Lake Basins.

	Healthy	Sub-Healthy	Moderate	Poor	Extremely Poor
B-IBI	IBI > 2.07	1.55 < IBI ≤ 2.07	1.04 < IBI ≤ 1.55	0.52 < IBI ≤ 1.04	IBI ≤ 0.52

**Table 7:** Results of ecosystem health assessment in different water sampling areas in Poyang Lake Basin.

Health Condition	YR		TJ		PY		NJ		JS		SH		Total	
	Sample Points	Proportion	Sample Points	Proportion	Sample Points	Proportion	Sample Points	Proportion	Sample Points	Proportion	Sample Points	Proportion	Sample Points	Proportion
Healthy	1	33.33%	1	16.67%	4	36.36%	3	60.00%	3	60.00%	1	20.00%	13	37.14%
Sub-healthy	—	—	—	—	4	36.36%	2	40.00%	2	40.00%	2	40.00%	10	28.57%
Moderate	—	—	5	83.33%	1	9.09%	—	—	—	—	2	40.00%	8	22.86%
Poor	1	33.33%	—	—	1	9.09%	—	—	—	—	—	—	2	5.71%
Extremely Poor	1	33.33%	—	—	1	9.09%	—	—	—	—	—	—	2	5.71%

## Data Availability

The datasets analyzed during the current study are available from the corresponding author upon reasonable request.

## Appendix A

**Table A1:** Candidate biological indicators and their responses to disturbance.

Parameter Attribute	Parameter Number	Biological Parameter	Parameter Description	Response to Disturbance
Species composition and abundance	M1	Total taxa	Number of benthic macroinvertebrate taxa in the sample	Decrease
	M2	EPT taxa	Number of Ephemeroptera + Plecoptera + Trichoptera taxa in the sample	Decrease
	M3	Crustacea + Mollusca taxa	Number of crustacean and mollusk taxa in the sample	Decrease
	M4	Ephemeroptera taxa	Number of Ephemeroptera taxa in the sample	Decrease
	M5	Coleoptera taxa	Number of Coleoptera taxa in the sample	Decrease
	M6	Trichoptera taxa	Number of Trichoptera taxa in the sample	Decrease
	M7	Diptera taxa	Number of Diptera taxa in the sample	Decrease
	M8	Chironomidae taxa	Number of Chironomidae taxa in the sample	Decrease
	M9	EPT (%)	(Ephemeroptera + Plecoptera + Trichoptera individuals)/Total individuals in the sample	Decrease
	M10	Crustacea + Mollusca (%)	(Crustacean + Mollusca individuals)/Total individuals in the sample	Decrease
	M11	Ephemeroptera (%)	Ephemeroptera individuals/Total individuals in the sample	Decrease
	M12	Coleoptera (%)	Coleoptera individuals/Total individuals in the sample	Decrease
	M13	Trichoptera (%)	Trichoptera individuals/Total individuals in the sample	Decrease
	M14	Diptera (%)	Diptera individuals/Total individuals in the sample	Increase
	M15	Chironomidae (%)	Chironomidae individuals/Total individuals in the sample	Increase
	M16	Oligochaeta (%)	Oligochaete individuals/Total individuals in the sample	Increase
Diversity	M17	Shannon–Wiener index	Calculated by formula	Decrease
	M18	Pielou index	Calculated by formula	Decrease
	M19	Richness index	Calculated by formula	Decrease
	M20	Simpson index	Calculated by formula	Decrease
Sensitivity and tolerance	M21	Sensitive taxa (TV ≤ 3)	Number of taxa with Tolerance Value (TV) ≤ 3	Decrease
	M22	Tolerant taxa (TV ≥ 7)	Number of taxa with Tolerance Value (TV) ≥ 7	Increase
	M23	Sensitive taxa (%)	(Individuals with TV ≤ 3)/Total individuals in the sample	Decrease
	M24	Tolerant Taxa (%)	(Individuals with TV ≥ 7)/Total individuals in the sample	Increase
	M25	Dominant Species (%)	(Individuals of most dominant species)/Total individuals in the sample	Increase
	M26	Top 3 Dominant Species (%)	(Individuals of top 3 dominant species)/Total individuals in the sample	Increase
Feeding Functional Groups	M27	Shredders (%)	Shredder individuals/Total individuals in the sample	Decrease
	M28	Collectors (%)	Collector individuals/Total individuals in the sample	Increase
	M29	Filterers (%)	Filterers individuals/Total individuals in the sample	Increase
	M30	Scrapers (%)	Scraper individuals/Total individuals in the sample	Decrease
	M31	Predators (%)	Predator individuals/Total individuals in the sample	Decrease

**Table A2:** Data on the diversity indices. C indicates spring, D indicates winter, Q indicates autumn, and X indicates summer.

Sample	Richness Index	Shannon–Weiner	Pielou Index
CYR1	0	0	0
CYR2	1	0	0
CYR3	3	1.09861228866811	1
DYR1	2	0.529706199057654	0.76420450650862
DYR2	0	0	0
DYR3	2	0.0358990284409962	0.051791350304557
QYR1	2	0.223718076065834	0.322756958897398
QYR2	2	0.636514168294813	0.918295834054489
QYR3	1	0	0
XYR1	0	0	0
XYR2	1	0	0
XYR3	2	0.500402423538188	0.721928094887362
CTJ4	4	1.09059947377948	0.786701226208884
CTJ5	3	1.09861228866811	1
CTJ6	1	0	0
CTJ7	2	0.410116318288409	0.591672778582327
CTJ8	4	1.33217904021012	0.960964047443681
CTJ9	4	1.242453325	0.896240625180289
DTJ4	2	0.25095480435762	0.362051251733998
DTJ5	2	0.244930026794635	0.353359335021421
DTJ6	9	1.58157310741415	0.719804941073228
DTJ7	1	0	0
DTJ8	4	0.914285581467099	0.659517637169433
DTJ9	10	1.40451606968384	0.609973578808134
QTJ4	3	0.362501021739676	0.329962649679761
QTJ5	1	0	0
QTJ6	0	0	0
QTJ7	9	1.48786138269121	0.677154897154393
QTJ8	3	0.955699891112534	0.869915529773626
QTJ9	12	2.04045519011617	0.821139574917332
XTJ4	4	0.199099914156536	0.143620229397526
XTJ5	6	1.53672246943722	0.857661140252986
XTJ6	1	0	0
XTJ7	4	1.03501663348432	0.746606682182711
XTJ8	2	0.562335144618808	0.811278124459133
XTJ9	8	1.47733677100331	0.71044881108313
CPY10	6	1.60520710745546	0.895883144486481
CPY11	3	0.955699891112534	0.869915529773626
CPY12	2	0.562335144618808	0.811278124459133
CPY13	6	1.69574253416963	0.946411928215015
CPY14	3	1.01140426470735	0.920619835714305
CPY15	5	1.47507631105469	0.916516443199602
CPY16	2	0.693147180559945	1
CPY17	1	0	0
CPY18	1	0	0
CPY19	1	0	0
CPY20	0	0	0
DPY10	10	1.90720474094698	0.828288494852995
DPY11	10	2.13425532309787	0.926895309794048
DPY12	10	1.48803119085618	0.646243735088764
DPY13	8	2.01615371726138	0.969564989854281
DPY14	4	1.11874333598575	0.80700273142711
DPY15	10	2.06751244168107	0.897909244688407
DPY16	0	0	0
DPY17	7	1.54278207838702	0.792833152720848
DPY18	1	0	0
DPY19	1	0	0
DPY20	9	1.91533317167766	0.871705692460301
QPY20	8	1.70355993434049	0.819239156376716
XPY20	7	1.73153540826228	0.889833176060516
QPY10	9	1.62297601153479	0.73864821478667
QPY11	5	1.16018624397852	0.720864243979353
QPY12	13	1.66666497996901	0.649784751157216
QPY13	14	2.070231994	0.784458893905427
QPY14	8	1.84074872856928	0.885213020743189
QPY15	6	1.58678470752805	0.885601407320416
QPY16	3	1.09861228866811	1
QPY17	8	1.80551494922852	0.868269154500956
QPY18	1	0	0
QPY19	6	0.926760564602932	0.51723491937353
XPY10	4	1.08325501051851	0.781403315846587
XPY11	6	1.4402347497046	0.803810318538513
XPY12	6	1.41324169041344	0.788745205304989
XPY13	14	2.39025733681791	0.905723915124794
XPY14	2	0.693147180559945	1
XPY15	3	0.735621939758795	0.669591945535779
XPY16	6	1.67698777432242	0.935944697445866
XPY17	3	1.05492016798614	0.960229717860761
XPY18	3	1.09861228866811	1
XPY19	3	0.974314752869349	0.886859507142915
CNJ21	11	1.77605476212089	0.740672364747694
CNJ22	8	1.36419303723545	0.656038176544945
CNJ23	4	0.659872013784827	0.475997040954392
CNJ24	8	1.51129077395926	0.726777234977422
CNJ25	9	0.80978214954624	0.368547738769593
DNJ21	8	1.27416326986915	0.612743010241028
DNJ22	10	1.89236951566801	0.821845638376544
DNJ23	6	0.866406431097394	0.483550636107797
DNJ24	9	1.24296164577051	0.565696223586485
DNJ25	10	1.59621685497326	0.693228172035848
QNJ21	6	0.874455398810259	0.488042850521114
QNJ22	4	0.470900127917266	0.339682639650109
QNJ23	8	1.31057323357431	0.630252501599822
QNJ24	7	1.05543195677292	0.542384733069668
QNJ25	6	1.19203251272669	0.665286012547351
XNJ21	5	0.970552590755782	0.603038230463906
XNJ22	5	1.47507631105469	0.916516443199602
XNJ23	4	1.27703425946614	0.921185496588554
XNJ24	3	0.259717624933192	0.236405170060547
XNJ25	4	0.478150579382401	0.344912734836587
DJS26	7	1.59453248461001	0.819427600695805
DJS27	6	1.51749809498804	0.846931812584097
DJS28	9	1.9415071796013	0.883617996825371
DJS29	3	0.283936266755864	0.258449927863169
DJS30	7	1.7388948450374	0.893615178420025
QJS30	3	0.858740913006287	0.781659664527667
QJS26	5	1.46481638489081	0.91014159264798
QJS27	6	1.44691898293633	0.807540860135486
QJS28	6	1.62602069242075	0.90749942743224
QJS29	4	1.21488965394912	0.876357639489852
XJS26	11	1.54803838135041	0.645582148191082
XJS27	9	1.36307341326878	0.620361444764691
XJS28	11	0.893435780026477	0.372591659928428
XJS29	9	1.47835813629106	0.672829783327534
XJS30	10	1.89026255383218	0.820930596477666
DSH31	5	1.08658415270008	0.675132693411418
DSH32	5	1.27801195876342	0.79407347676467
DSH33	8	1.7003183727299	0.817680294756605
DSH34	4	0.851108979568139	0.613945352039511
DSH35	9	1.73972154347212	0.791781396138056
QSH31	11	2.06528328861355	0.861290028819042
QSH32	7	1.82387331598544	0.937285473777337
QSH33	14	2.40919349182936	0.912899263230706
QSH34	7	1.83437197028162	0.94268071481726
QSH35	5	1.08701095739633	0.675397882079428
